# Analyzing *Medicago* spp. seed morphology using GWAS and machine learning

**DOI:** 10.1038/s41598-024-67790-4

**Published:** 2024-07-30

**Authors:** Jacob Botkin, Cesar Medina, Sunchung Park, Kabita Poudel, Minhyeok Cha, Yoonjung Lee, Louis K. Prom, Shaun J. Curtin, Zhanyou Xu, Ezekiel Ahn

**Affiliations:** 1 Department of Plant Pathology, University of Minnesota, St. Paul, MN 55108 USA; 2 Department of Agronomy and Plant Genetics, University of Minnesota, St. Paul, MN 55108 USA; 3 Sustainable Perennial Crops Laboratory, United States Department of Agriculture- Agricultural Research Service, Beltsville Agricultural Research Center, Beltsville, MD 20705 USA; 4 Department of Biotechnology, Korea University, Seoul, 02841 Republic of Korea; 5 United States Department of Agriculture- Agricultural Research Service, Southern Plains Agricultural Research Center, 2765 F & B Road, College Station, TX 77845 USA; 6 Plant Science Research Unit, United States Department of Agriculture- Agricultural Research Service, St. Paul, MN 55108 USA; 7 Center for Plant Precision Genomics, University of Minnesota, St. Paul, MN 55108 USA; 8 Center for Genome Engineering, University of Minnesota, St. Paul, MN 55108 USA

**Keywords:** *Medicago sativa*, Alfalfa, Seed morphology, Area size, Seed color, RGB, GWAS, Machine learning, Plant development, Plant genetics

## Abstract

Alfalfa is widely recognized as an important forage crop. To understand the morphological characteristics and genetic basis of seed morphology in alfalfa, we screened 318 *Medicago* spp., including 244 *Medicago sativa* subsp. *sativa* (alfalfa) and 23 other *Medicago* spp., for seed area size, length, width, length-to-width ratio, perimeter, circularity, the distance between the intersection of length & width (IS) and center of gravity (CG), and seed darkness & red–green–blue (RGB) intensities. The results revealed phenotypic diversity and correlations among the tested accessions. Based on the phenotypic data of *M. sativa* subsp. *sativa*, a genome-wide association study (GWAS) was conducted using single nucleotide polymorphisms (SNPs) called against the *Medicago truncatula* genome. Genes in proximity to associated markers were detected, including CPR1, MON1, a PPR protein, and Wun1(threshold of 1E−04). Machine learning models were utilized to validate GWAS, and identify additional marker-trait associations for potentially complex traits. Marker S7_33375673, upstream of Wun1, was the most important predictor variable for red color intensity and highly important for brightness. Fifty-two markers were identified in coding regions. Along with strong correlations observed between seed morphology traits, these genes will facilitate the process of understanding the genetic basis of seed morphology in *Medicago* spp.

## Introduction

Seed morphology is a valuable tool for assessing seed quality, particularly in seed maturity and viability, which significantly impacts seed germination^1^ and subsequent crop yield^2^. The "Queen of the forages," alfalfa (*Medicago sativa* L., 2n = 4x = 32), is an essential source of nutrient-rich livestock feed, and had an economic value of 11 billion dollars in the United States during 2022^3,4^. Alfalfa exhibits diverse seed morphological characteristics, including shape, size, and color. For example, the color of alfalfa seeds varies from yellow or olive green to brown to black and white^5^. Multiple studies have revealed that seed color affects both seed vigor and germination^6–8^. However, depending on the plant species, the relationship between seed vigor and seed color was found to vary^9^. Seed color also depicts information on the other agronomic characteristics of the plant, such as salt stress tolerance and seedling performance of alfalfa^9^. Both seed size & shape are also used as parameters in the identification/classification of taxa to evaluate plant genetic diversity^10,11^. Prom et al.^12^ reported that seed weight is associated with biotic stress and germination rate in sorghum. Similarly, Ahn et al.^13^ surveyed Senegalese sorghum seed morphological characteristics such as seed color, shape, and size and identified strong correlations between the seed morphological traits. Likewise, a deep understanding of the genetic diversity of alfalfa accessions is crucial to selecting genetically diverse parents and broadening the genetic basis of the cultivated alfalfa. Moreover, integrating advanced techniques such as Genome-Wide Association Studies (GWAS) with traditional methods has enabled a more comprehensive analysis, thereby enhancing understanding of the intricate relationship between seed characteristics and their genetic determinants^14^. To have a comprehensive idea of alfalfa seed morphology, 244 alfalfa germplasms in addition to 23 other *Medicago* spp. were evaluated for various seed morphological traits such as seed area, length, width, length-to-width ratio (LWR), perimeter, circularity, the distance between the intersection of length and width (IS) and center of gravity (CG), and seed darkness & red–green–blue (RGB) intensities (86,294 seeds for seed size-related traits and 25,440 seeds for color-related traits). We analyzed correlations within the collection to explore potential relationships between seed morphological traits. Building upon the phenotypic data collected of each trait, we combined it with 8,565 single nucleotide polymorphisms (SNPs) across the *Mt5.0* reference genome of *M. truncatula* to conduct GWAS^15^. Tracing the top candidate SNPs to the reference genome, we identified candidate genes possibly directly connected with seed morphology. Furthermore, validation of the GWAS results and identification of additional marker-trait associations within coding sequences and the entire genome was accomplished using machine learning models.

## Materials and methods

### Seed phenotypic evaluation in *Medicago* spp.

The plant Medicago spp. genetic resources used in the described research were provided, free of cost, by the USDA ARS National Plant Germplasm System, Plant Germplasm Introduction and Testing Research Unit following request submission and justification through the publicly accessible GRIN-Global online platform^16^. Overall, we obtained 318 accessions of *Medicago* spp., including 244 *Medicago sativa* subsp. *sativa* (alfalfa) and 23 other species from the germplasm collection (complete list available in Supplementary Data S1).

Seeds were evaluated for seed area size (mm^2^), length (mm), width (mm), LWR, perimeter (mm), circularity (0–1 range, 0: not circular to 1: complete circle), the distance between the intersection of length & width (IS) (points where the width and length hit the boundary for the seed parameter) and center of gravity (CG) (the point where the seed’s mass is concentrated) and seed darkness & brightness/RGB values (0–255 range, 0: absence of light and highest intensity of RGB colors and darkness to 255: full light), following the methodology of previous studies^13,17^. Briefly, seed images (~ 100 seeds per accession) were acquired with Canon imageRUNNER ADVANCE C7270 (Canon Inc, Tokyo, Japan), followed by analysis through the SmartGrain (version 1.3) high-throughput phenotyping software. The seed image analysis was conducted for seed area size, length, width, LWR, perimeter, circularity, and distance between IS and CG^17^. For every image, any errors produced by the SmartGrain program were fixed manually. Seed darkness and brightness/RGB values were obtained using a multi-point function in ImageJ version 1.54d^18^. Overall, 86,294 seeds for seed size-related traits and 25,440 seeds for color-related traits were analyzed.

### Statistical analysis

Statistical analysis followed the methods described by Ahn et al.^13^. To compare all possible accession pairs for each trait, Tukey’s HSD test was employed using JMP Pro 15 (SAS Institute, Cary, NC, USA). Additionally, one-way ANOVA was performed for each trait. To explore potential correlations between the traits, Pearson’s correlation coefficients were calculated for all possible pairs using JMP Pro 15. We also utilized principal component analysis (PCA) and clustering variables analysis with the phenotypic data from all traits using JMP Pro 15.

### SNP identification

For the genotyping of each accession, we obtained genome sequencing reads from 189 phenotyped accessions generated through genotyping-by-sequencing (GBS), sourced from the NCBI SRA database under the Bioproject PRJNA287263^19^. The *Mt5.0* reference genome of *Medicago truncatula* (GCF_003473485.1) was downloaded from NCBI (https://www.ncbi.nlm.nih.gov/datasets/genome/). The fastq reads were aligned to the reference genome using the Bwa-mem2 program with the ‘mem’ method. Subsequently, the alignments (BAM files) were merged into a single BAM file using the SamTools v1.17^20^. SNP variants were called as diploid using freebayes v1.3.6^21^, with the following parameters: —min-base-quality 10, —min-supporting-allele-qsum 10, —read-mismatch-limit 3, —min-coverage 5, —min-alternate-count 4, —mismatch-base-quality-threshold 10. The SNPs were further filtered based on the following criteria: SNPs should be biallelic, have a missing genotype rate of 10% or less, exhibit a minor allele frequency of at least 0.05, and have a read supporting the allele of at least 5. Finally, a single variant call format file with 8565 SNPs for 189 accessions was generated for downstream analyses. This population with 189 accessions exhibited an expected heterozygosity of 0.285. For accessions with available genotype data, the population structure and genetic diversity was evaluated by Zhang et al.^19^.

### Genome-wide association study

We conducted genome-wide association analysis using the R-package, Genome Association and Prediction Integrated Tool (GAPIT) version 3^22^ by employing the method known as settlement of mixed linear models under progressively exclusive relationship (SUPER)^23^. SUPER method uses the framework of a standard MLM approach. An MLM can be described using Henderson’s matrix notation as follows:

1 $$\mathbf{y}=\mathbf{W}\mathbf{v}+\mathbf{X}{\varvec{\upbeta}}+\mathbf{Z}\mathbf{u}+\mathbf{e}$$

where **y** is the vector of observed phenotypes; **v** is the marker effect; **β** is an unknown vector containing fixed effects, including the population stratification (**Q**) and the intercept; **u** is a vector of size *n* (number of individuals) of random additive genetic effects; **W**, **X,** and **Z** are the known design matrices for marker, fixed and random effects, respectively; and **e** is the unobserved vector of residuals. The **u** and **e** vectors are assumed to be normally distributed with zero mean and **G** and **R** covariance matrices, respectively. $\mathbf{G}=2\mathbf{K}{\sigma }_{a}^{2}$, where **K** is a known kinship matrix with element ${K}_{i,j}(i,j=1, 2,\dots ,n)$ calculated from genetic markers, and $\mathbf{R}=\mathbf{I}{\sigma }_{e}^{2}$, where **I** is the identity matrix and is the unknown residual variance. Population stratification was corrected by principal component analysis (PCA). The optimal number of principal components was determined through a Bayesian information criterion-based analysis within GAPIT. For the association analysis in GAPIT, the following parameters were used: PCA.total = 3 and model = SUPER while leaving all other settings at their default values. Significant associations of SNPs with the traits of interest were identified using a threshold of − log10(*p*-value) > 4. Subsequently, the associated regions in the genome were defined as extending 10 kb upstream and downstream of the significant SNPs. Within these regions, candidate genes were identified based on the gene annotation of the *Mt5.0* reference genome of *Medicago truncatula* (GCF_003473485.1).

### Machine learning

The VCF file was converted to numeric format using the `gt2num` function from the bwardr R package (v1.0, https://github.com/etnite/bwardr). One hundred eighty-eight monomorphic markers were removed using the `nearZeroVar` function from the R package caret v.6.0–94^24^. The genotypic matrix was merged with 11 phenotypic traits to run three machine learning methods: random forest (RF), support vector machine with the linear kernel (SVM), and extreme gradient boosting with the linear kernel (XGB). All models were trained using a resampling with ten-fold cross-validation, and the root mean squared error (RMSE) was used to select the optimal model based on the smallest value. Importance scores for predictor variables (markers) were calculated on trained models using the `varImp` function, and the values were scaled from 0 to 100.

## Results

### Seed morphologies

**Figure 1:**
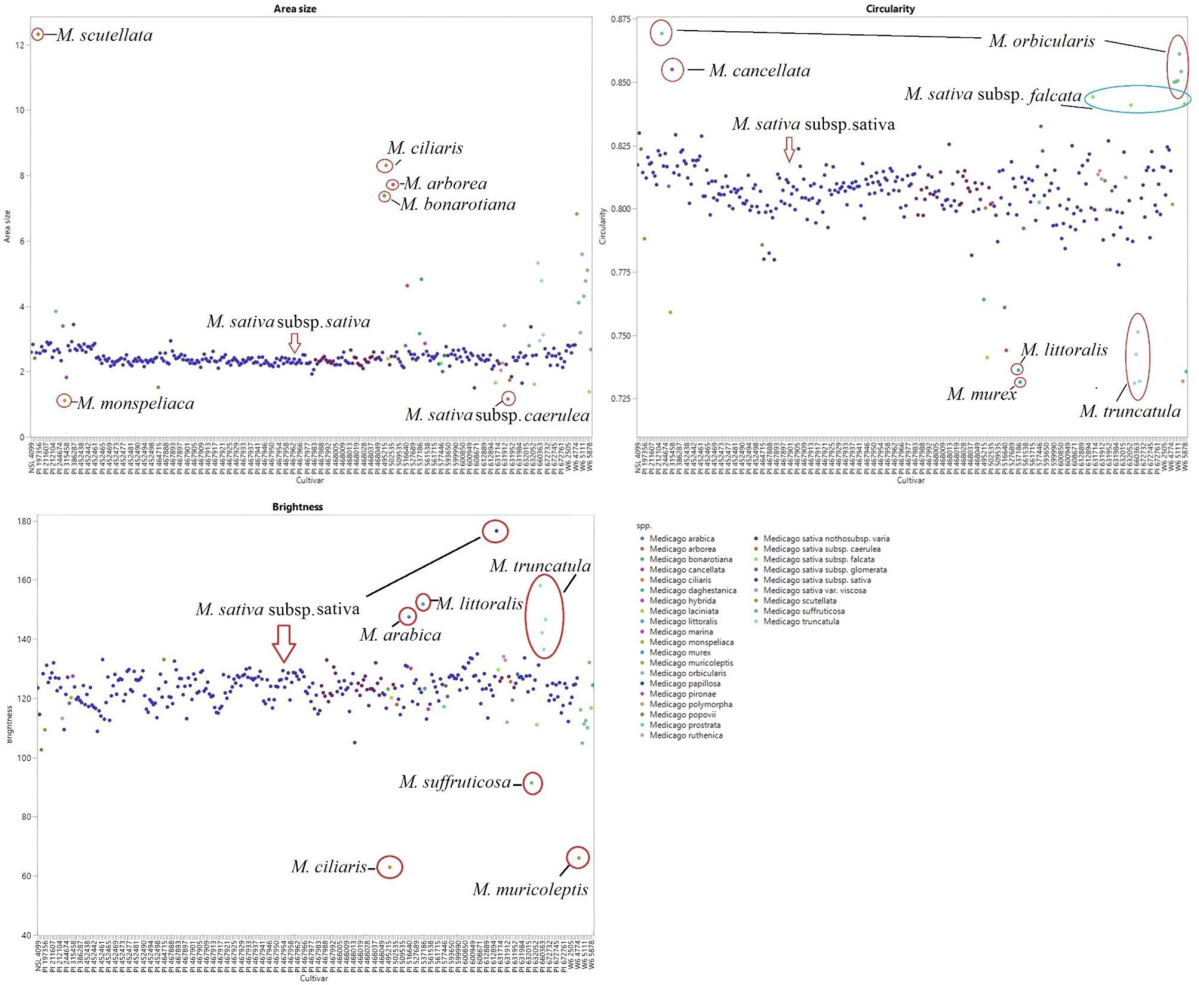
Scatter plots of seed area, circularity, and brightness across *Medicago* spp. (**a**) *M. scutellata* has the largest seed area, followed by *M. ciliaris*, *M. arborea*, and *M. bonarotiana*. Conversely, *M. sativa* subsp. *caerulea* and *M. monspeliaca* exhibit the smallest seed area across tested *Medicago* spp. (**b**) *M. orbicularis* displays the most circular shape followed by *M. cancellata* and *M. sativa* subsp. *falcata*. In contrast, *M. truncatula*, *M. murex*, and *M. littoralis* have the least circular shapes. (**c**) PI 619,434 (*M. sativa* subsp. *sativa*) is the brightest accession across all *Medicago* spp., followed by *M. truncatula*, *M. littoralis*, *M. arabica*, and other *M. sativa* subsp. *sativa* accessions. *M. ciliaris* and *M. muricoleptis* present the darkest seeds.

**Figure 2:**
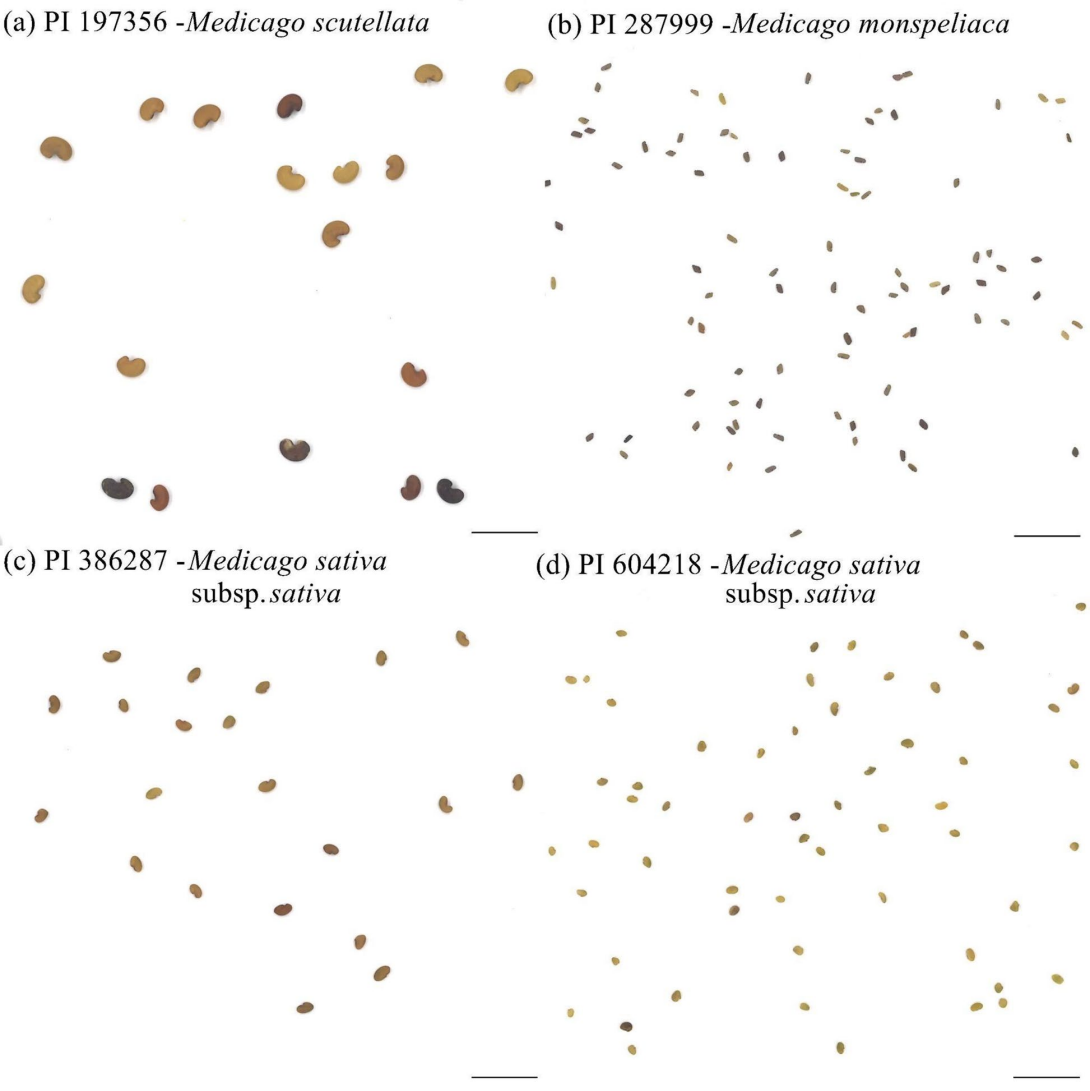
Comparison of seed area sizes for accessions PI 197,356, PI 287,999, PI 386,287, and PI 604,218. (**a**) PI 197,356 (*Medicago scutellata*) has the largest seed area size among all evaluated *Medicago* spp. (**b**) PI 287,999 (*Medicago monspeliaca*) exhibits the smallest seed area size of all evaluated *Medicago* spp. (**c**) PI 386,287 has the largest seeds in area size in *Medicago sativa* subsp. *sativa*. (**d**) PI 604,218 has the smallest seeds in area size in *Medicago sativa* subsp. *sativa*. The scale bars indicate 1 cm.

**Figure 3:**
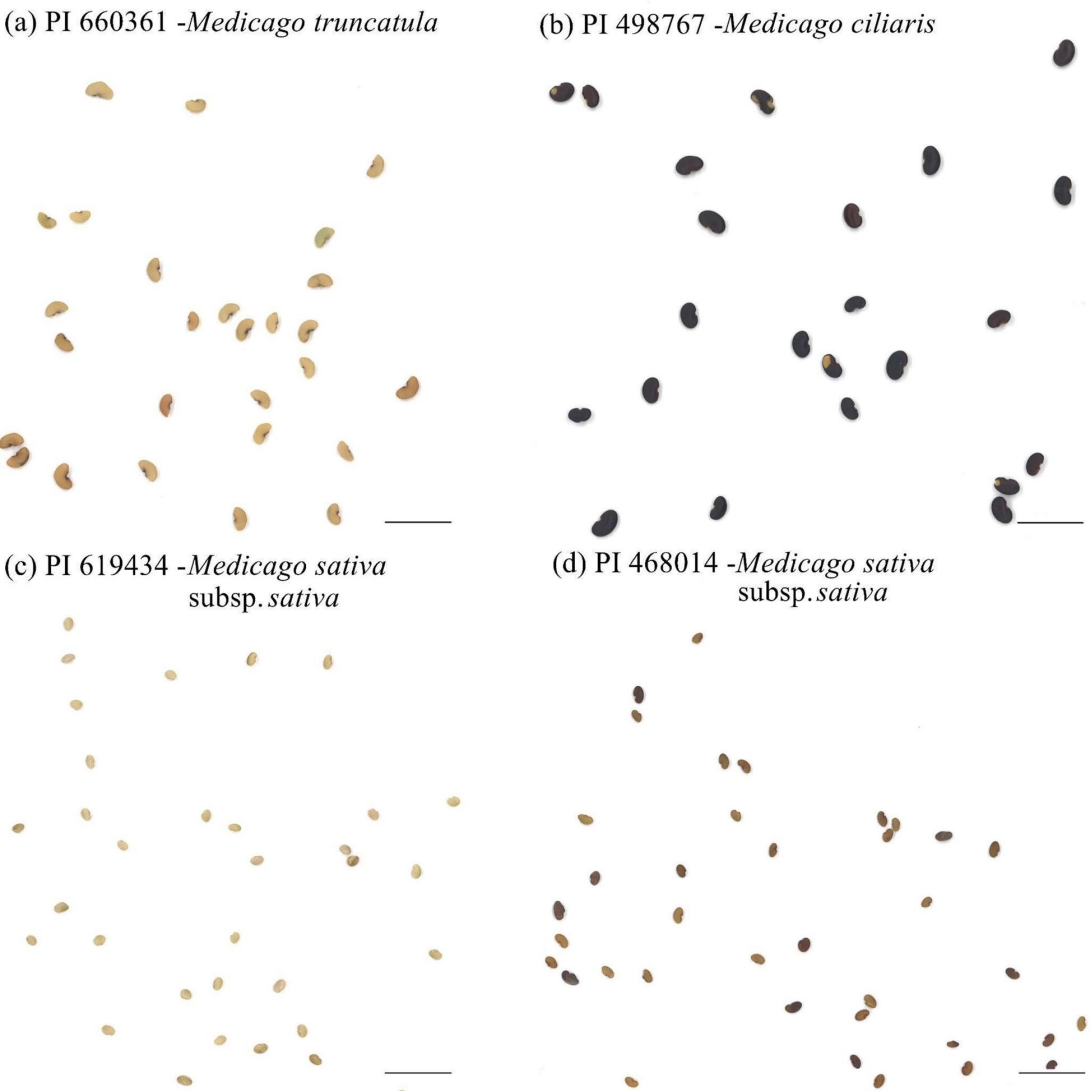
Comparison of seed brightness for PI 660,361, PI 498,767, PI 619,434, and PI 468,014. (**a**) PI 660,361 (*Medicago truncatula*) has the second brightest seeds across all evaluated *Medicago* spp. accessions (**b**) PI 498,767 (*Medicago ciliaris*) possesses the darkest seeds in the population of *Medicago* spp. (**c**) PI 619,434 has the brightest seeds in all evaluated *Medicago* spp. (**d**) PI 468,014 has the darkest seeds in *Medicago sativa* subsp. *sativa*. The scale bars indicate 1 cm.

Two-tailed ANOVA detected significant differences (*p* < 0.0001) for all evaluated seed traits among the *Medicago* spp. accessions (raw data available in Supplementary Data S1). The extensive morphological variations are shown in Fig. 1, presented in scatter plots of seed area, circularity, and brightness across *Medicago* spp., and highlight both the substantial diversity between species and the wide range within alfalfa itself. Further illustrating this point, Table 1 details the top five accessions for each trait (detailed quantitative morphological data available in Supplementary Data S1). For instance, the area size for PI 386,287 (largest in alfalfa) is 3.44 ± 0.48 mm^2^, while that for PI 604,218 (smallest in alfalfa) was 1.5 ± 0.34 mm^2^ (Table 1 and Fig. 1). Figure 2 visually emphasizes the variation among the *Medicago* spp., presenting the seed area sizes of four select accessions: PI 197,356, PI 287,999, PI 386,287, and PI 604,218. Similarly, Fig. 3 captures the contrasts in seed colors observed across the population, featuring examples like PI 660,361, PI 498,767, PI 619,434, and PI 468,014. The population also displayed notable variation in other traits as well.

**Table 1:** The top five accessions for each seed morphological trait in *M. sativa* subsp. *sativa* are displayed.

Largest area size (mm^2^)		Smallest area size (mm^2^)	
Accession	Mean ± S.D	Accession	Mean ± S.D
1. PI 386,287	3.44 ± 0.48	1. PI 604,218	1.5 ± 0.34
2. PI 632,044	3.37 ± 0.49	2. PI 632,004	1.65 ± 0.26
3. PI 467,891	2.94 ± 0.42	3. PI 467,982	1.92 ± 0.33
4. PI 352,685	2.92 ± 0.43	4. PI 467,983	2.06 ± 0.36
5. PI 211,608	2.9 ± 0.46	5. PI 618,621	2.13 ± 0.3
Longest perimeter (mm)		Shortest perimeter (mm)	
1. PI 632,044	7.32 ± 0.6	1. PI 604,218	4.76 ± 0.57
2. PI 386,287	7.28 ± 0.56	2. PI 632,004	5.15 ± 0.46
3. PI 467,891	6.87 ± 0.54	3. PI 467,982	5.44 ± 0.49
4. PI 352,685	6.69 ± 0.54	4. PI 467,983	5.62 ± 0.52
5. PI 211,608	6.67 ± 0.58	5. PI 618,621	5.63 ± 0.48
Longest length (mm)		Shortest length (mm)	
1. PI 632,044	2.7 ± 0.24	1. PI 604,218	1.72 ± 0.24
2. PI 386,287	2.65 ± 0.23	2. PI 632,004	1.9 ± 0.18
3. PI 467,891	2.54 ± 0.22	3. PI 467,982	1.99 ± 0.21
4. PI 452,440	2.45 ± 0.19	4. PI 618,621	2.06 ± 0.21
5. PI 211,610	2.44 ± 0.24	5. PI 467,983	2.06 ± 0.21
Longest width (mm)		Shortest width (mm)	
1. PI 386,287	1.7 ± 0.13	1. PI 632,004	1.15 ± 0.1
2. PI 632,044	1.64 ± 0.14	2. PI 604,218	1.17 ± 0.14
3. PI 26,590	1.59 ± 0.14	3. PI 467,983	1.35 ± 0.12
4. PI 352,685	1.59 ± 0.13	4. PI 618,621	1.32 ± 0.1
5. PI 452,445	1.57 ± 0.14	5. PI 595,589	1.33 ± 0.15
Highest LWR		Lowest LWR	
1. PI 467,891	1.7 ± 0.18	1. PI 346,817	1.47 ± 0.15
2. PI 467,888	1.7 ± 0.14	2. PI 468,013	1.48 ± 0.13
3. PI 467,885	1.7 ± 0.15	3. PI 619,434	1.48 ± 0.15
4. PI 600,865	1.69 ± 0.19	4. PI 26,590	1.48 ± 0.15
5. PI 509,535	1.68 ± 0.18	5. PI 604,218	1.48 ± 0.21
Highest circularity (0–1 scale)		Lowest circularity (0–1 scale)	
1. PI 26,590	0.83 ± 0.03	1. PI 632,004	0.78 ± 0.04
2. PI 346,817	0.83 ± 0.03	2. PI 467,891	0.78 ± 0.04
3. PI 452,462	0.83 ± 0.03	3. PI 467,885	0.78 ± 0.03
4. PI 468,013	0.83 ± 0.03	4. PI 467,888	0.78 ± 0.03
5. PI 604,218	0.82 ± 0.04	5. PI 600,865	0.78 ± 0.04
Longest distance between IS and CG (mm)		Shortest distance between IS and CG (mm)	
1. PI 600,774	0.25 ± 0.17	1. PI 467,977	0.12 ± 0.09
2. PI 467,891	0.25 ± 0.16	2. PI 467,983	0.13 ± 0.09
3. W6 2516	0.24 ± 0.16	3. PI 468,013	0.13 ± 0.09
4. PI 632,044	0.24 ± 0.15	4. PI 452,494	0.13 ± 0.09
5. PI 599,990	0.23 ± 0.16	5. PI 467,982	0.13 ± 0.1
Brightest (0–255 scale)		Darkest (0–255 scale)	
1. PI 619,434	176.54 ± 16.87	1. PI 468,014	105.01 ± 17.14
2. PI 604,218	134.98 ± 12.5	2. PI 452,444	108.83 ± 13.45
3. PI 632,004	133.65 ± 9.23	3. PI 234,481	109.41 ± 15.57
4. PI 600,865	133.63 ± 9.99	4. W6 2502	112.14 ± 11.95
5. PI 600,949	133.39 ± 9.97	5. PI 452,466	112.54 ± 10
Highest red intensity (0–255 scale)		Lowest red intensity (0–255 scale)	
1. PI 619,434	207.81 ± 15.34	1. PI 468,014	134.03 ± 26.7
2. PI 467,890	174.13 ± 9.16	2. PI 452,444	143.94 ± 19.21
3. PI 600,865	173.5 ± 13.14	3. PI 234,481	144.69 ± 21.59
4. PI 604,218	172.96 ± 16.65	4. W6 2502	145.81 ± 16.1
5. PI 452,462	172.8 ± 14.53	5. PI 26,590	148.08 ± 16.06
Highest green intensity (0–255 scale)		Lowest green intensity (0–255 scale)	
1. PI 619,434	184.29 ± 18.54	1. PI 468,014	104.66 ± 20.79
2. PI 604,218	147 ± 15.93	2. PI 452,444	109.63 ± 16.33
3. PI 632,004	143.7 ± 11.97	3. W6 2502	109.96 ± 15.73
4. PI 600,865	143.66 ± 12.87	4. PI 234,481	110.78 ± 19.74
5. PI 600,949	141.36 ± 12.4	5. PI 612,890	111.83 ± 14.87
Highest blue intensity (0–255 scale)		Lowest blue intensity (0–255 scale)	
1. PI 619,434	137.43 ± 17.53	1. PI 234,481	72.91 ± 7.12
2. PI 641,418	89.81 ± 7.96	2. PI 452,444	73.01 ± 6.64
3. PI 600,949	86.98 ± 5.88	3. PI 537,440	73.25 ± 6.0
4. PI 601,132	86.34 ± 4.93	4. PI 511,303	73.26 ± 4.71
5. PI 452,477	86.11 ± 7.12	5. PI 452,493	73.76 ± 6.1

### Correlations among the traits

**Figure 4:**
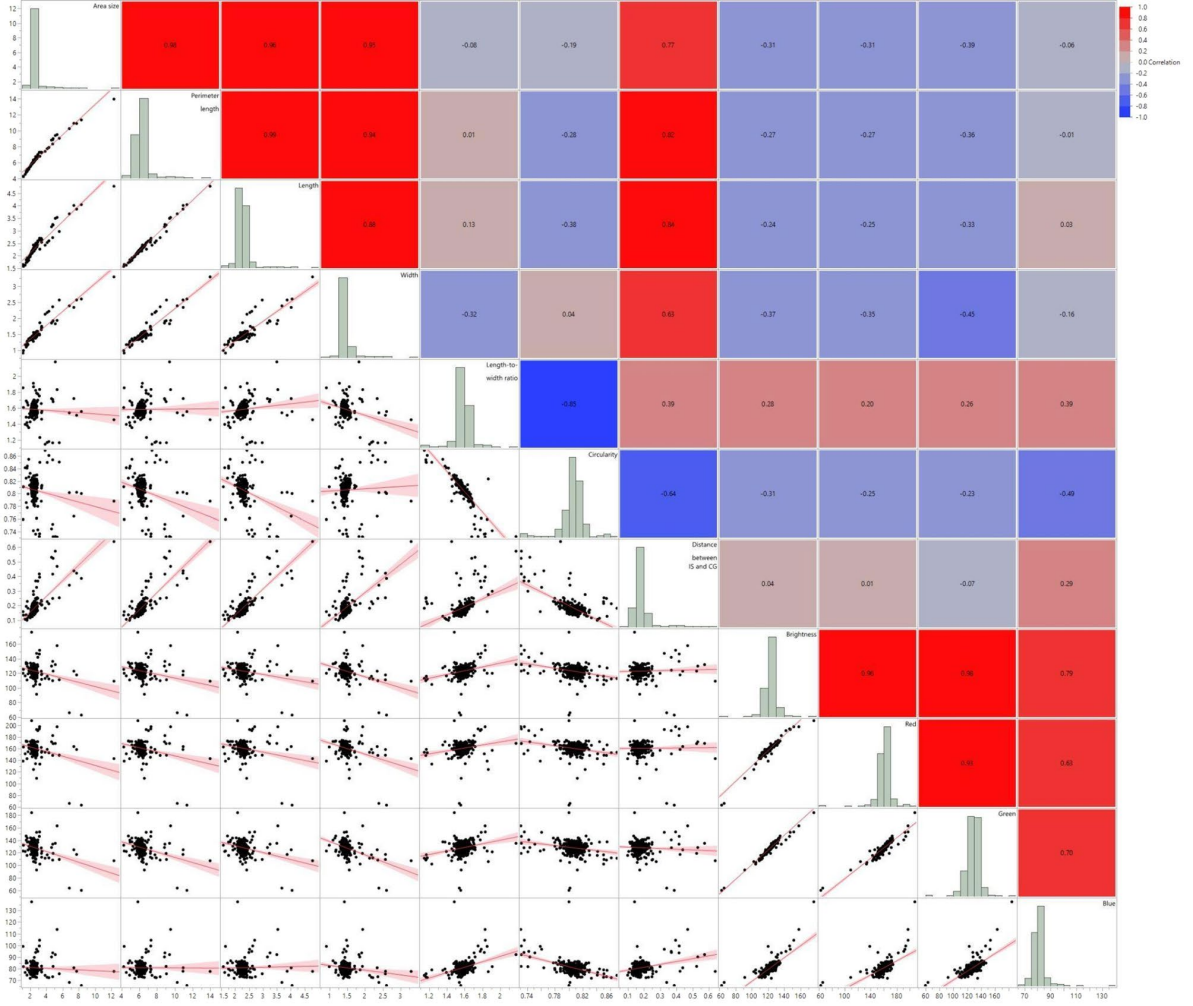
Scatter plots, histograms, and heatmaps reveal correlations among seed morphology traits based on Pearson’s r.

**Figure 5:**
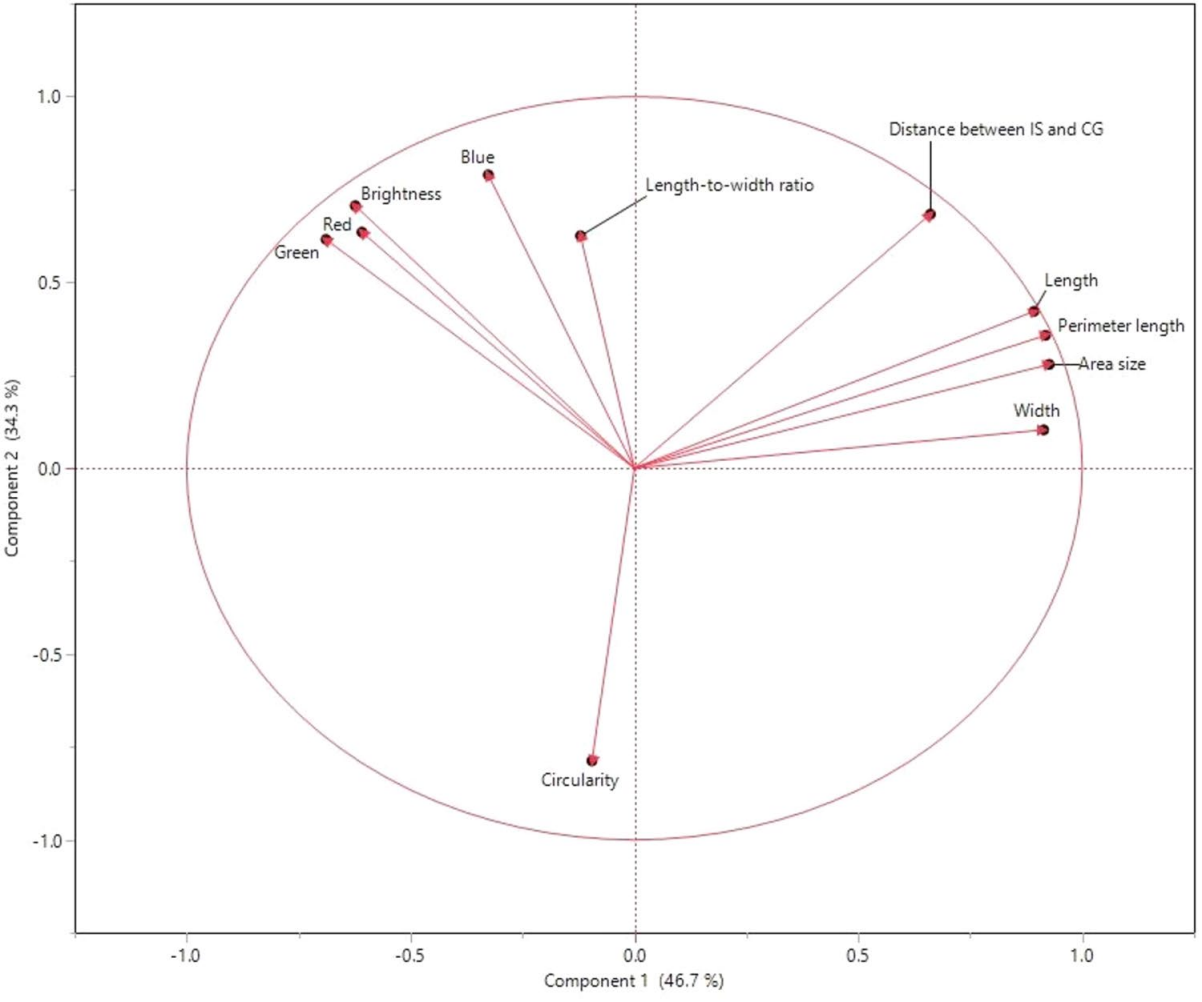
Biplot of the PCA of seed morphological traits in *Medicago* spp. PC1 and PC2 are displayed, representing the two principal components that explain 81% of the variance in the data.

**Figure 6:**
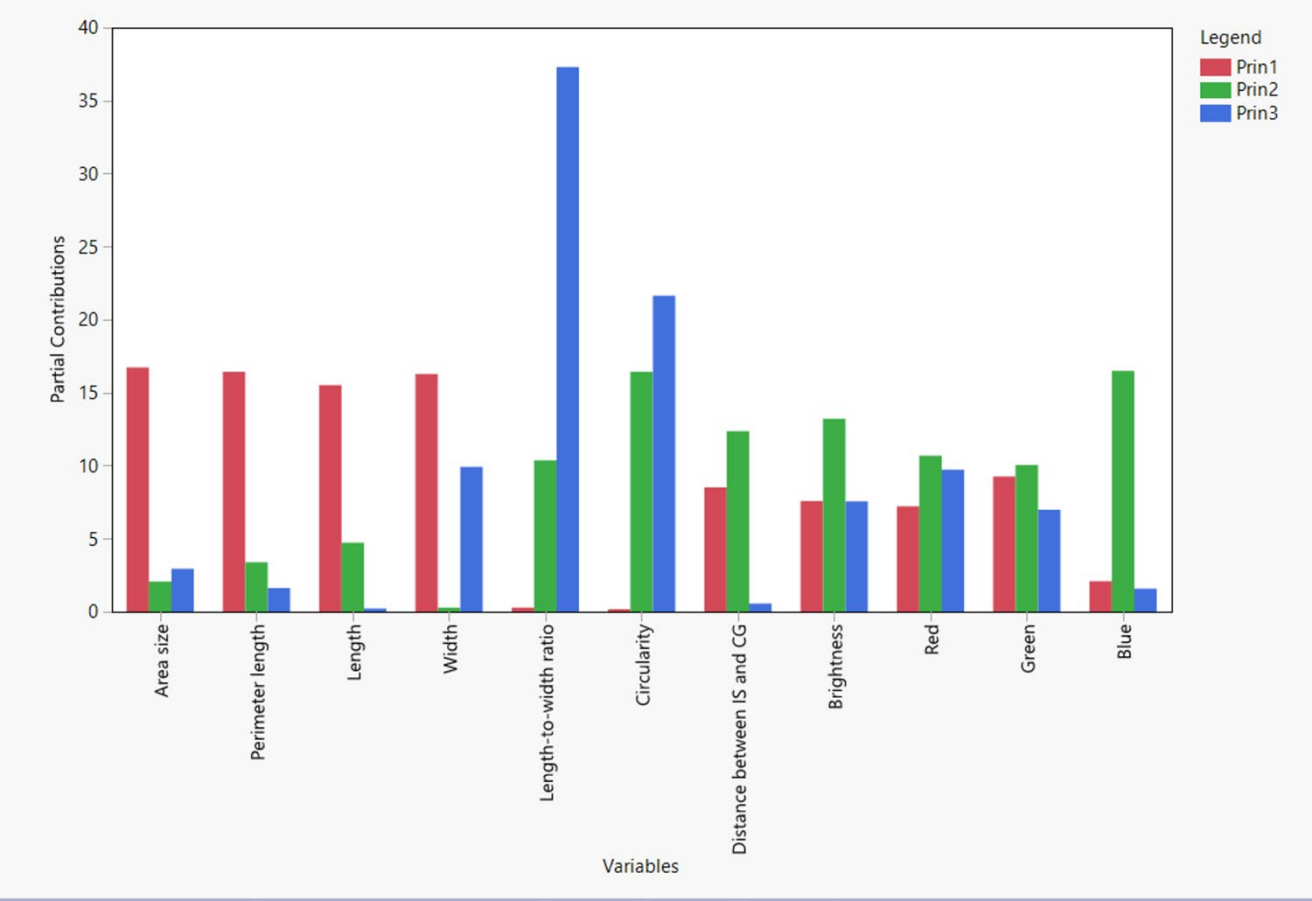
Partial contribution of seed morphology-related traits to principal components in *Medicago* spp. The chart displays the partial contribution of each seed morphology-related trait to the first three principal components (PC1, PC2, and PC3).

Pearson’s correlation analysis found intercorrelations among most evaluated seed morphology traits, with a few notable exceptions. Blue color intensity stood apart, showing no significant correlations with area, perimeter, and length (Fig. 4 & Table 2). Similarly, IS and CG displayed no significant correlation with brightness, red, and green color intensity. Brightness, red, and green color intensities show moderate correlations with the three seed shape-related parameters: Area, perimeter, and length, indicating a meaningful separation between blue and other colors, as shown in Fig. 5. A PCA plot encompassing all traits unveiled that two major PCs (PC1 & PC2) capture 81% of the overall variation (Fig. 5). Circularity was distinctly separated from the other two major groups. The partial contribution of variables plotted in Fig. 6 indicates that PC1 mainly comprises area size, perimeter, length, and width. Both color intensity- and seed morphology-related traits contributed to PC2. A majority of PC3 consists of LWR and circularity. Likewise, clustering analysis identified three clusters based on seed size, color, and shape (Table 3).

**Table 2:** Correlations between the seed morphology-related traits.

	Area	Perimeter	Length	Width	LWR	Circularity	IS and CG	Brightness	Red	Green	Blue
Area (mm^2^)	1.00***	0.98***	0.96***	0.95***	− 0.08	− 0.19**	0.77***	− 0.31***	− 0.31***	− 0.39***	− 0.06
Perimeter (mm)	0.98***	1.00***	0.99***	0.94***	0.01	− 0.28***	0.82***	− 0.27***	− 0.27***	− 0.36***	− 0.01
Length (mm)	0.96***	0.99***	1.00***	0.88***	0.13*	− 0.38***	0.84***	− 0.24***	− 0.25***	− 0.33***	0.03*
Width (mm)	0.95***	0.94***	0.88***	1.00***	− 0.32***	0.04	0.63***	− 0.37***	− 0.35***	− 0.45***	− 0.16***
LWR	− 0.08	0.01	0.13*	− 0.32***	1.00***	− 0.85***	0.39***	0.28***	0.20**	0.26***	0.39***
Circularity	− 0.19**	− 0.28***	− 0.38***	0.04	− 0.85***	1.00***	− 0.64***	− 0.31***	− 0.25***	− 0.23***	− 0.49***
IS and CG	0.77***	0.82***	0.84***	0.63***	0.39***	− 0.64***	1.00***	0.04	0.01	− 0.07	0.29***
Brightness	− 0.31***	− 0.27***	− 0.24***	− 0.37***	0.28***	− 0.31***	0.04	1.00***	0.96***	0.98***	0.79***
Red	− 0.31***	− 0.27***	− 0.25***	− 0.35***	0.20**	− 0.25***	0.01	0.96***	1.00***	0.93***	0.63***
Green	− 0.39***	− 0.36***	− 0.33***	− 0.45***	0.26***	− 0.23***	− 0.07	0.98***	0.93***	1.00***	0.70***
Blue	− 0.06	− 0.01	0.03	− 0.16**	0.39***	− 0.49***	0.29***	0.79***	0.63***	0.70***	1.00***

*** = *p* < 0.0001, ** = *p* < 0.001 and * = *p* < 0.01.

**Table 3:** Cluster variables analysis among the seed morphology-related traits.

Cluster	Members	R^2^ with its own cluster	R^2^ with the next closest	1−R^2^
1	Perimeter length	0.994	0.062	0.006
	Length	0.969	0.072	0.034
	Area size	0.967	0.086	0.036
	Width	0.864	0.129	0.156
	Distance between IS and CG	0.723	0.289	0.39
2	Brightness	0.996	0.094	0.004
	Green	0.939	0.115	0.069
	Red	0.901	0.063	0.106
	Blue	0.677	0.207	0.406
3	Length-to-width ratio	0.926	0.087	0.081
	Circularity	0.926	0.111	0.083

Three clusters were formed based on seed characteristics: Size, color, and shape.

### Genome-wide association studies

Because of the mixed ploidy among different species of Medicago included in this study, the SNP variants were called diploid, and genome-wide association studies (GWAS) were conducted on 189 accessions using 8,565 biallelic SNPs. GWAS identified five SNPs exceeding the significance threshold of − log10(*p-value*) = 4. Five different markers were associated with three different traits (Table 4). Markers S2_9100992 and S2_9101010, separated by 18 bp, were associated with the trait distance between IS and CG (IS to CG). These two markers were located in the gene MtrunA17_Chr2g0289631. Two markers (S4_29996363 and S6_30969602) were associated with seed width. Marker S4_29996363 was in a region coding for a vacuolar fusion protein, MON1, and marker MtrunA17_Chr6g0476151 was in a region coding for a tetratricopeptide-like helical domain protein. Finally, marker S7_33375673, associated with red color intensity (red) was located 99 bp upstream of the coding region for a wound-induced protein, Wun1. Manhattan plots for these three traits are presented in Supplementary Fig. 1. The PCA plot based on GBS data showed a single main cluster, indicating limited population differentiation (Supplementary Fig. 2). Linkage disequilibrium plots of top SNPs show that linkage was significant (R^2^ > 0.3) in a continuous region of approximately 9 kb surrounding marker S4_29996363, the top SNP for seed width (Supplementary Fig. 3. Interestingly, this SNP occurred in the intronic region of MON1, which occupies the majority (~ 7.1 kb) of the linked region.

**Table 4:** List of the top SNPs and candidate genes identified by GWAS.

Marker	Trait	− Log_10_(*p-value*)	Gene ID	Protein
S2_9100992	IS to CG	4.3	MtrunA17_Chr2g0289631	F-box protein CPR1
S2_9101010	IS to CG	4.2	MtrunA17_Chr2g0289631	F-box protein CPR1
S4_29996363	Width	4.7	MtrunA17_Chr4g0029011	Vacuolar fusion protein, MON1
S6_30969602	Width	4.1	MtrunA17_Chr6g0476151	Tetratricopeptide-like helical domain
S7_33375673	Red	4.6	MtrunA17_Chr7g0244221*	Wound-induced protein, Wun1

Candidate genes (Gene ID) were identified based on the gene annotation of the reference genome^15^. Traits associated were the distance between IS and CG (IS to CG), seed width (Width), and red color intensity (Red).

Gray cells correspond to markers at the same gene.

*Marker was located at 99 bp upstream of the coding region.

### Machine learning

**Figure 7:**
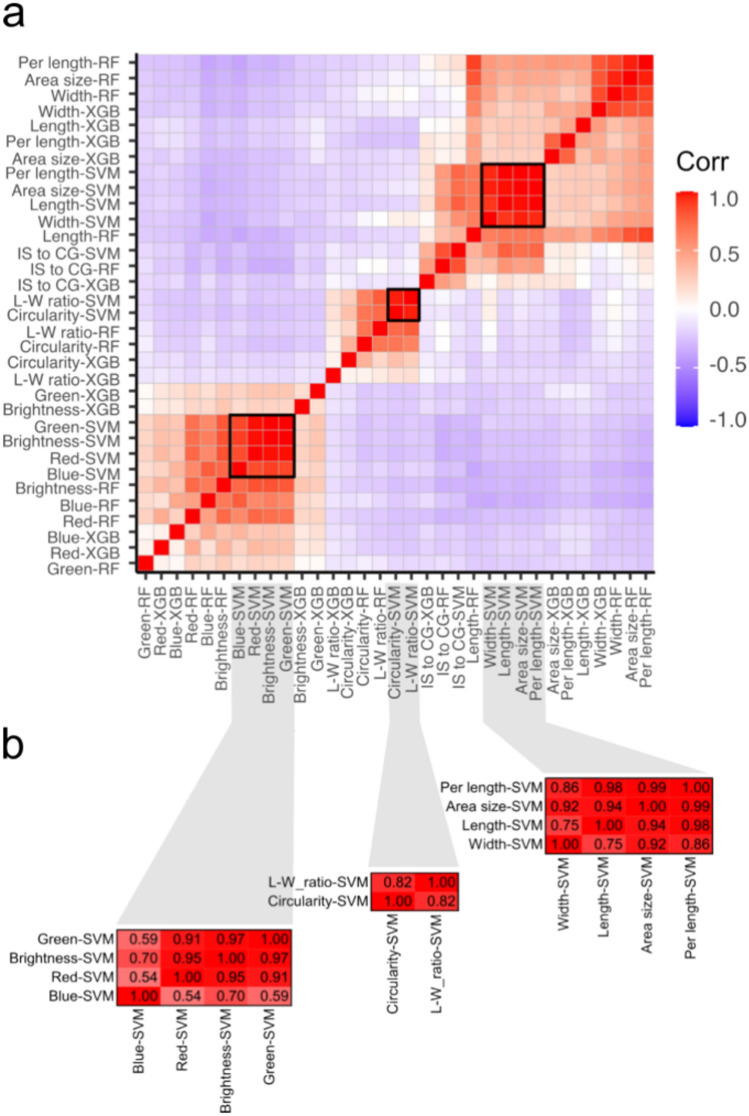
Pearson correlation of predictor variable importance for trained machine learning models. (**a**) The relative importance of 8377 predictor variables (markers) was calculated for 11 phenotypic traits using three machine learning models: random forest (RF), support vector machine (SVM), and extreme gradient boosting (XGB). (**b**) Clusters of highly correlated traits with correlation values. Pearson correlation scores were colored with red indicating 1 and blue indicating − 1.

The importance scores predictor variables (markers) among three machine learning models, and 11 phenotypic traits were compared using Pearson correlation, identifying three clusters of traits highly correlated by their importance scores calculated with SVM (Fig. 7a). Cluster one groups importance scores for brightness, blue, green, and red color intensity; cluster two groups length-to-width ratio and circularity; and cluster three groups area size, length, perimeter length, and width (Fig. 7b). The importance scores for predictor variables (markers) were calculated and compared with markers associated with GWAS. Marker S7_33375673, associated with red color intensity (Red) by GWAS, was the most important predictor variable for the RF and SVM models (Table 5). The importance scores of the other markers were moderate, averaging around 38.8. However, importance scores for markers associated with GWAS were similar across different traits. For instance, marker S7_33375673 had an importance score of 89.3 and 89.2 with RF and SVM for brightness.

**Table 5:** Comparison of the relative importance of markers associated with GWAS.

Marker	Trait	GWAS	RF	SVM	XGB
S2_9100992	IS to CG	4.3	23.3	24.2	–
	L-W ratio	–	19.0	40.7	–
	Circularity	–	17.9	39.2	–
S2_9101010	IS to CG	4.2	26.5	26.3	10.1
	L-W ratio	–	12.6	39.4	–
	Circularity	–	14.2	36.7	–
S4_29996363	Width	4.7	–	56.5	20.4
	Length	–	20.2	56.6	–
	Area size	–	–	58.9	–
	Per length	–	–	63.3	–
S6_30969602	Width	4.1	–	27.9	–
	Blue	–	–	31.3	–
	Area size	–	–	21.9	–
S7_33375673	Red	4.6	100.0	100.0	15.3
	Brightness	–	89.3	89.2	77.6
	Green	–	30.6	69.5	–
	Blue	–	51.6	54.5	–

Importance scores for markers associated with GWAS were calculated using three machine learning models: random forest (RF), support vector machine (SVM), and extreme gradient boosting (XGB). The importance scores for predictor variables were scaled from 0 to 100, and only values greater than 10 were included. GWAS values correspond to − log10(*p-values*). Traits tested included distance between IS and CG (IS to CG), length-to-width ratio (L–W ratio), perimeter length (Per length), and blue, green, or red color intensity (Blue, Green, or Red).

The relative importance of predictor variables was filtered to retain markers with an importance score greater than 70. In total, 301 markers were selected, with the number of markers per trait ranging from 9 to 49. Among these, 117 markers were identified uniquely, while 184 markers were identified multiple times by different models and/or for different traits (Supplementary Table 1). By model, SVM had the highest number of markers retained (182), while XGB had the lowest (54), and by trait, circularity had the highest number of markers retained (43), while width had the lowest (6) (Supplementary Table 2). Twenty-nine markers with relative importance > 70 by more than one model were selected as highly important predictor variables. For example, marker S8_46680823 had a relative importance greater than 70 for area size, length, distance between IS and CG, and perimeter length, identified by different models (Supplementary Table 3). Interestingly, marker S8_46680823 is located in the coding region of an RNA helicase (MtrunA17_Chr8g0389051). Fifty-two unique markers were located in coding regions and were annotated using *Mt5.0* reference genome of *Medicago truncatula* (Supplementary Table 4), identifying six transcription factors, four genes with glycosylation signaling function, and eight related to plant development.

## Discussion

Studies emphasize the importance of seed size and shape in breeding for improved yield and quality. In plant breeding, it is crucial to determine the size and shape of seeds, as well as their correlation, to enhance yield or quality^10^. The priority in crop domestication has been on large seeds for a long time, as they have been established as crucial components for high yield^25,26^. Pattern analysis showed that seed weight significantly correlated with multiple traits such as seed perimeter, length, width, and thickness in *Sophora moorcroftiana*^27^. Along with seed size and weight, seed color plays an important role in crops. Research in wheat indicates that variations in seed color may arise from genetic diversity^28^. Brown seeds’ germination parameters and seedling performance in alfalfa were significantly lower than green and yellow seeds under different salt stress^9^. Also, brown seeds were less resistant to salt stress^9^. In addition, seed shape is an important trait often used in plant identification and classification^10^. A recent study in sorghum identified potential correlations between seed shape (circularity) and seed size (area size, length, and width)^13^.

Alfalfa [*Medicago sativa* (L.) subsp. *sativa*], a member of the Fabaceae, has been cultivated as a forage crop since the beginning of recorded history^29^. Though extensively studied for traits like flowering time^30^, forage quality^31^, abiotic stress response^32^, and leaf size^33^, alfalfa’s seed morphology-associated traits and their correlations have received less attention. Given alfalfa’s critical role as a forage crop, deciphering the genetic mechanisms shaping seed architecture necessitates a deep understanding of seed morphology and its link to molecular markers. To address this gap, we explored 11 essential seed morphology traits in 318 accessions of *Medicago* spp., focusing on alfalfa. We identified significant phenotypic variations (Figs. 1, 2, 3 and Table 1) and numerous correlations among traits (Figs. 4, 5, 6 and Tables 2, 3). Cluster analysis of seed morphology-related traits (Table 3) revealed three distinct clusters representing seed size, color, and shape. The partial contribution of these traits to principal components (Fig. 6) further highlighted similar patterns of correlations. Notably, these results closely align with sorghum seed morphology, suggesting the universality of correlations between seed morphology-associated traits across diverse plant species. We detected deviations of blue color intensity from brightness, red, and green, but the underlying cause (genetic diversity or technological limitations) remains unclear.

The GWAS analysis revealed five markers with − log10(*p-values*) greater than 4 associated with three traits. For seed width, GWAS identified markers S4_29996363 and S6_30969602. Marker S4_29996363 was located in a gene coding for a vacuole fusion protein, MON1. Vacuoles, which can occupy up to 90% of plant cell volume, play crucial roles in plant growth, development, and various functions such as storage, transport, and stress response^34^. MON1 interacts with calcium caffeine zinc sensitivity1 protein in processes such as vacuolar trafficking, vacuole biogenesis, and plant growth^35^. Marker S6_30969602 was located within a gene annotated as a tetratricopeptide-like helical domain protein. While much has been learned about the molecular functions of these proteins over the past decade, including their roles within the cell and their physiological functions during plant growth and development^36^, there are no reports of this gene being associated with seed width. Additionally, the GWAS identified the marker S7_33375673 associated with seed red intensity. This marker was located 99 bp upstream of a gene annotated as Wound-induced protein, Wun1. Wun1 plays an important role in plant responses to diverse stresses, including wounding (biotic) and high salt (abiotic)^37^.

While several advances have been made to increase statistical power and reduce computing time in GWAS, all models are based on mixed linear models (MLMs). In this study, we employed the Settlement of MLM Under Progressively Exclusive Relationship (SUPER) method for GWAS. The SUPER method divides the genome into equal-sized segments to select the most influential SNP, considered as a pseudo quantitative trait nucleotide (QTN). These pseudo QTNs are exempt from the kinship matrix (K). Since the K is derived from a smaller number of markers compared to the K in MLM, which is derived from all markers, the confounding between K and some markers becomes more severe^23^. However, despite this method, we only identified five markers associated with three out of 12 traits. Several possible explanations exist: 1) MLMs in GWAS test each SNP separately to capture the main genetic effect, neglecting the minor effects of markers, 2) GWAS cannot capture SNP-SNP interactions as it only tests for the marginal effects of SNPs, overlooking the contribution of multiple markers to phenotypic response, and 3) GWAS requires control of type I errors through stringent methods such as Bonferroni or false discovery rate corrections, which may lead to underpowered detection of candidate markers.

On the other hand, machine learning (ML) models applied to GWAS can enhance the detection of markers associated with traits of interest. ML has the capability to capture the combined minor effects of multiple genetic markers, allowing for the development of multi-locus methodologies that consider all SNPs in the model to estimate the importance scores of predictor variables (SNPs)^38^. In this study, we implemented three ML models, confirming the significance of marker S7_33375673 not only for seed red intensity but also for seed brightness. Additionally, we identified 29 markers with relative importance greater than 70 by more than one model, thereby expanding the repertoire of markers and candidate genes related to seed-related traits. Among the ML models, Support Vector Machines (SVM) exhibited lower shrinkage of estimated importance for predictor variables, representing 60% of the SNPs with an importance score greater than 70. In summary, machine learning can enhance the ability to detect new genetic associations with various traits, addressing the challenges posed by the complex genetic architecture of quantitative traits.

The identified candidates are promising leads due to their known functions related to plant development. However, the GWAS analysis conducted in this study was limited by the availability of genotypic data due to the relatively low sequencing data coverage. In *M. truncatula,* the mean linkage disequilibrium has been shown to extend to less than 5 kilobases^39^. We identified 8,565 SNPs, meaning variants with potentially significant associations with seed morphology traits could have been missed in our analysis. While selfing was used to produce the seeds, their tetraploid nature may still introduce some degree of genetic heterogeneity compared to seeds from diploid plants, which could reduce the power of the GWAS analysis. To address this potential limitation, we employed a larger sample size (~ 100 seeds per accession) to increase the statistical power of the analysis. On the other hand, machine learning models identify highly important SNPs related to their phenotypic traits. This highlights the importance of including machine learning models in association studies. Future studies will investigate the associations between seed morphology traits and molecular markers through machine learning to expand the identification of genetic targets in alfalfa to improve the yield and quality of this vital forage crop.

## Conclusion

In this study, we explored seed morphological diversity and correlations between multiple traits among *Medicago* spp., focusing primarily on alfalfa. We evaluated seed morphology traits in a diverse panel of 318 *Medicago* spp. accessions and present a comprehensive set of phenotype data. High phenotypic diversity and extensive correlations were identified among the tested accessions across the traits. PCA and clustering variables were generated to uncover commonalities between phenotypes, mainly revealing three groups: Seed size, shape, and color. Among seed colors, blue intensity exhibited minor differences from the others. GWAS analysis based on morphological traits identified multiple SNPs potentially associated with seed morphologies. Candidate genes for seed morphology trait IS and CG, seed width, and red intensity included CPR1, MON1 and a PPR protein, and Wun1, respectively. Some GWAS-associated markers were further validated with ML models, such as marker S7_33375673, which was upstream of wound-induced protein 1 (Wun1). Finally, a number of additional markers were identified using ML models, which shows the value of applying algorithms to assess marker association with complex traits. Further research is therefore warranted to elucidate the specific genes underlying seed morphological diversities in *Medicago* spp.

### Supplementary Information

Supplementary Figures.

Supplementary Tables.

Supplementary Information 3.

## Data Availability

Data Availability Statement GBS raw data were submitted to the NCBI Sequence Read Archive with bio- project ID: PRJNA287263 and biosample accession numbers: AMN03779142—SAMN03779330. The vcf file, and genotypic matrix for machine learning methods, R script, and phenotypic data are available in figshare (https://doi.org/10.6084/m9.figshare.26236538.v1).
