# Supplementary material for: Analyzing Medicago spp. seed morphology using GWAS and machine learning
Source: Sci Rep. 2024 Jul 30;14:17588. doi: 10.1038/s41598-024-67790-4 (PMC11289399; doi:10.1038/s41598-024-67790-4)
Supplement: Supplementary file 1 — Supplementary Figures. [file 41598_2024_67790_MOESM1_ESM.docx]

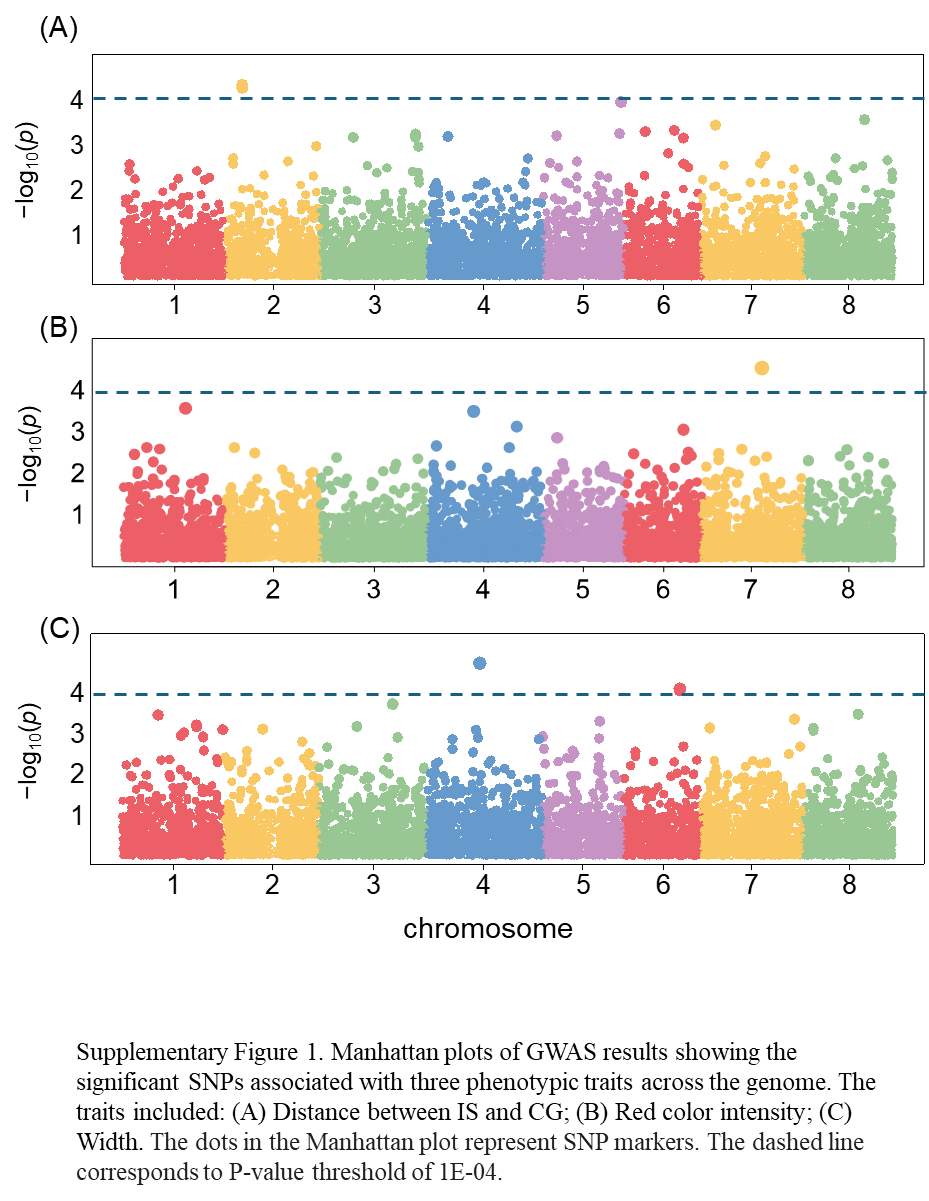


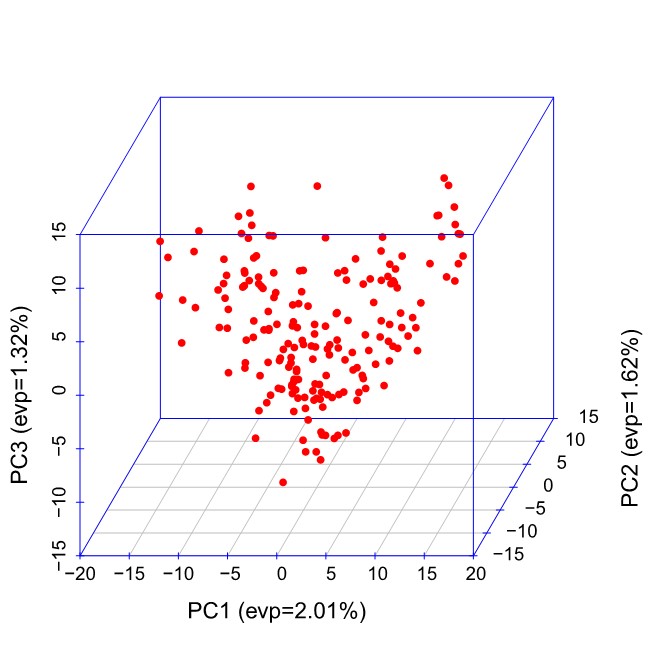
Supplementary Figure 2. The principal component analysis of the alfalfa accessions based on GBS presenting PC1, PC2, and PC3. One group formed without any separate clustering among the accessions.


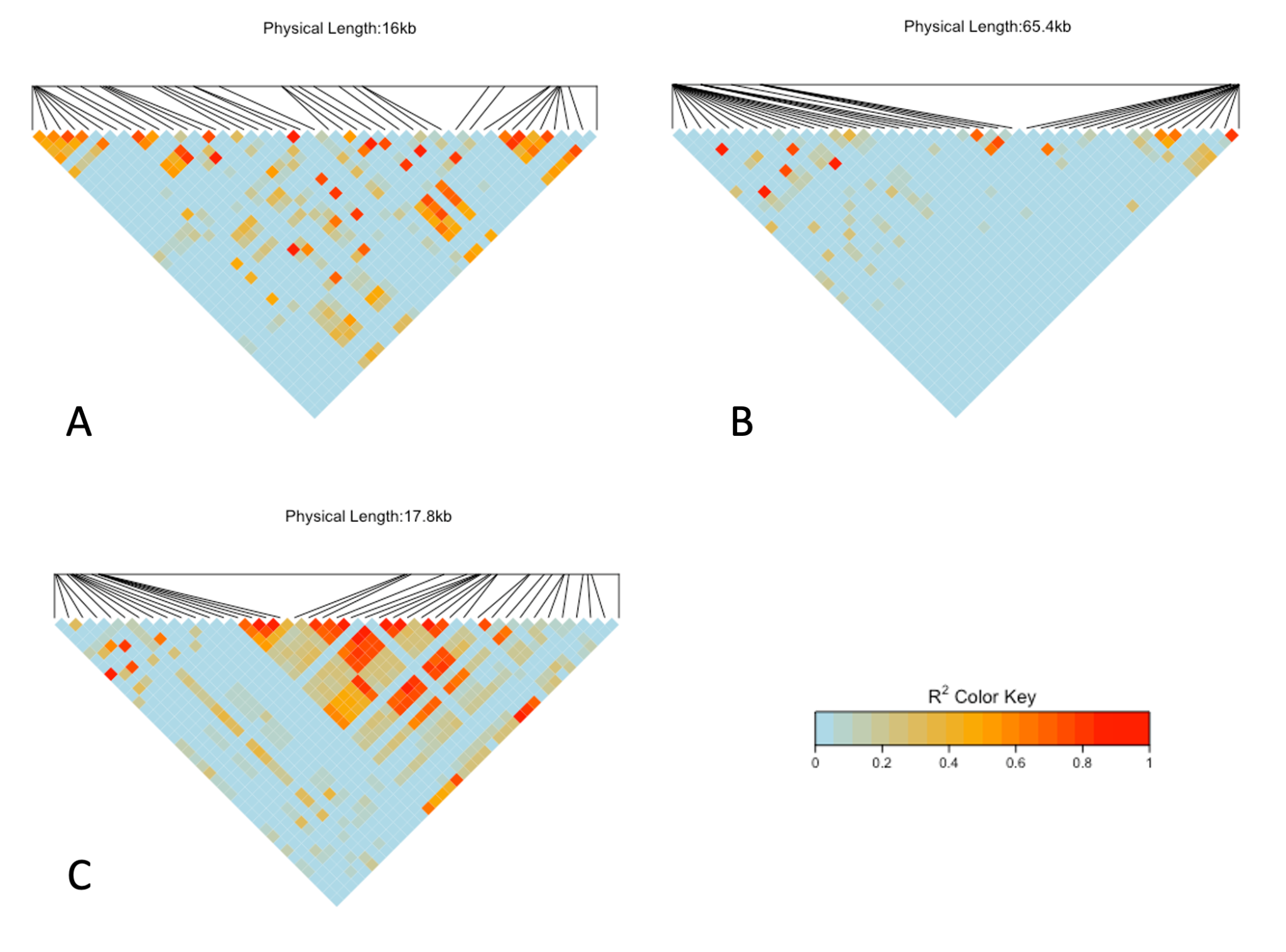


Supplementary Figure 3. Linkage disequilibrium plots showing correlations of the ten nearest markers up and downstream of the most significant SNP for phenotypes (A) IS and CG (markers 2_9101010 and 2_9100992) (B) Red intensity (marker 7_33375673) and (C) Width (marker 4_29996363).
